# Association between the composite dietary antioxidant index and urinary phthalate concentrations: Evidence from NHANES 2011–2018

**DOI:** 10.1097/MD.0000000000049659

**Published:** 2026-07-10

**Authors:** Yongjiao Yang, Pan Ding, Jiancheng Pan, Yuhong Feng, Zhongcheng Xin, Changli Wu

**Affiliations:** a Department of Urology, The Second Hospital of Tianjin Medical University, Tianjin City, China; b Institute of Urology, Tianjin Medical University, Tianjin City, China.

**Keywords:** CDAI, cross-sectional study, endocrine disrupting chemicals, NHANES, phthalates

## Abstract

The relationship between the Composite Dietary Antioxidant Index (CDAI) and urinary phthalate concentrations has yet to be explored. This study utilized data from the 2011–2018 NHANES database. Weighted multivariable linear regression models were employed to examine the relationship between CDAI and urinary phthalate concentrations. Subgroup analyses were conducted to explore differences in associations across various subgroups. Additionally, restricted cubic spline (RCS) analyses were performed to investigate potential nonlinear relationships between CDAI and urinary phthalate concentrations. In the multivariable linear regression model, fully adjusted for confounding variables, CDAI was significantly associated with lower concentrations of high-molecular-weight (HMW) phthalates, including MCOP (*β* = −0.724, 95% CI: −1.454, −0.006) and MECP (*β* = −0.297, 95% CI: −0.618, −0.091). MBzP also showed a significant negative association with CDAI (*β* = −0.212, 95% CI: −0.353, −0.071). For low-molecular-weight (LMW) phthalates, there was a significant association between CDAI and lower MiBP concentrations (*β* = −0.136, 95% CI: −0.318, −0.046). Subgroup analyses indicated a negative correlation between CDAI and urinary phthalate concentrations in participants aged 60 years and older, as well as in male participants. Weighted RCS analysis demonstrated a linear relationship between CDAI and urinary phthalate concentrations. CDAI negatively correlated with urinary phthalate concentrations; further research is needed for validation.

## 1. Introduction

Phthalates, a prevalent class of synthetic chemicals, are extensively employed as plasticizers in a wide array of everyday consumer products.^[1,2]^ The widespread use of phthalates has raised substantial concerns regarding their potential health risks.^[3,4]^ Studies have demonstrated that phthalates possess endocrine-disrupting properties,^[5-7]^ which may adversely impact reproductive system development,^[8,9]^ induce metabolic disorders, and contribute to other health complications.^[10]^ Human exposure to phthalates primarily occurs through dietary intake, inhalation, and dermal contact.^[11,12]^ Once inside the body, phthalates are metabolized by enzymes into monoesters and other metabolites, which are predominantly excreted through the kidneys into the urine.^[13]^ Consequently, the concentration of phthalates in urine serves as a reliable biomarker for assessing exposure levels. Given the widespread use of phthalates and their potential health risks, understanding the factors influencing phthalate exposure is crucial for developing effective public health interventions.

Diet is a significant source of phthalate exposure, as these chemicals can leach into food from packaging materials and processing equipment.^[14]^ Among various dietary components, certain nutrients have garnered attention for their potential protective effects against oxidative stress and inflammation induced by environmental pollutants.^[15]^ Therefore, dietary patterns and nutrient intake may play a critical role in modulating phthalate exposure levels. The Composite Dietary Antioxidant Index (CDAI) serves as an integrative metric for assessing the antioxidant capacity of a diet by amalgamating various antioxidant nutrients, including vitamins A, C, and E, zinc, selenium, and carotenoids, to evaluate the overall dietary antioxidant potential.^[16]^ Previous studies have indicated that increased intake of dietary antioxidants is associated with reduced oxidative stress and inflammation,^[17]^ which are key pathways through which phthalates exert their toxic effects.^[18,19]^ However, the relationship between CDAI and urinary phthalate concentrations has yet to be explored.

This study utilizes data from the 2011–2018 National Health and Nutrition Examination Survey (NHANES) to investigate the association between the CDAI and urinary phthalate concentrations. Our findings will contribute to the understanding of dietary factors influencing phthalate exposure and provide insights for public health strategies aimed at mitigating the adverse effects of phthalates through dietary modifications.

## 2. Materials and methods

### 2.1. Data source and study population

**Figure 1.**
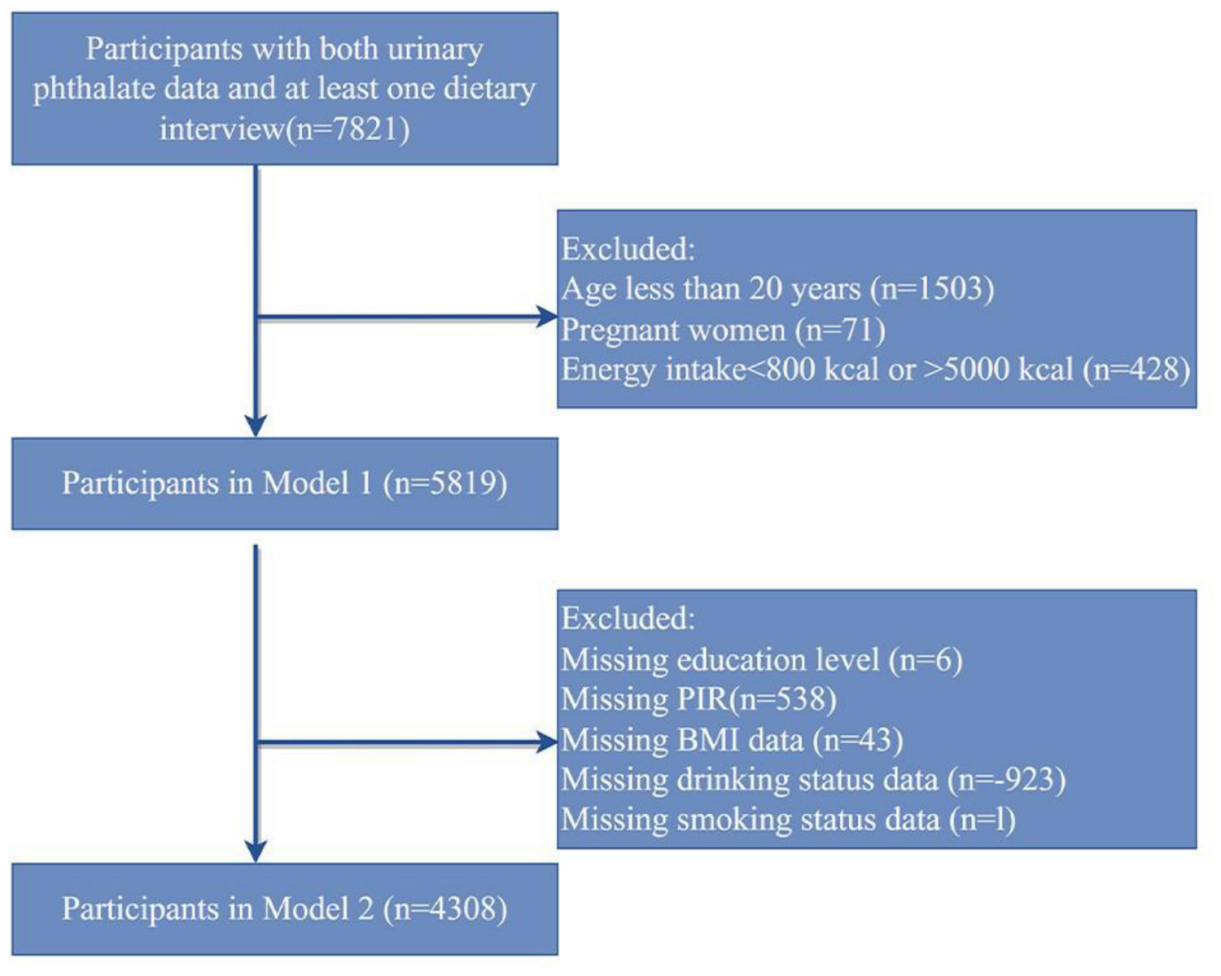
The flowchart for participant screening in this study. BMI = body mass index, PIR = poverty income ratio.

This study was approved by the Ethics Committee of The Second Hospital of Tianjin Medical University. This study utilized data from the 2011 to 2018 NHANES. NHANES is an ongoing cross-sectional survey designed to assess the health and nutritional status of adults and children in the United States. The survey employs a complex, multistage probability sampling design to obtain a representative sample. The participant selection process for this study is illustrated in Figure 1. For this analysis, we initially identified 7821 participants who had both urinary phthalate data and at least 1 day of dietary interview data. After excluding participants who were under 20 years of age, pregnant women, and those with an average daily energy intake of <800 kcal or more than 5000 kcal, a total of 5819 participants were included in the model 1 analysis. Missing data were handled using a complete-case approach. Participants with missing information on key covariates, including education level, economic status, BMI, alcohol consumption, and smoking status, were excluded from the fully adjusted model. Therefore, model 1 included 5819 participants, whereas model 2 included 4308 participants after the exclusion of missing covariate data.

### 2.2. Assessment of CDAI

The CDAI was calculated to assess the overall antioxidant capacity of the diet. Dietary antioxidant intake data were collected by trained interviewers using two 24-hour dietary recalls. The CDAI is derived from the intake levels of several key antioxidants, including vitamins A, C, and E, carotenoids, selenium, and zinc.^[16]^ According to the measurement method described by Wright et al,^[20]^ each individual’s intake of dietary antioxidants was adjusted by subtracting the population mean intake and then dividing by the population standard deviation to weight the results. The individual scores for each dietary antioxidant were then summed to generate a total CDAI score for each participant, as shown in the following formula:


$$CDAI=\sum_{i=1}^{n=6}\frac{Individual\;Intake-Mean}{SD}.$$

CDAI was calculated using antioxidant nutrient intake from 24-hour dietary recall data, including vitamins A, C, and E, carotenoids, selenium, and zinc. Each nutrient was standardized by subtracting the population mean and dividing by the population standard deviation, and the standardized values were summed to obtain the CDAI score. Dietary supplement intake was not included in the primary CDAI calculation. Energy-adjusted CDAI was not used in the main analysis; therefore, total energy intake was considered in sensitivity analyses/acknowledged as a limitation.

### 2.3. Assessment of urinary phthalate concentrations

High-performance liquid chromatography-tandem mass spectrometry (HPLC-MS/MS) was employed for the quantitative detection of phthalate concentrations in urine. Urine samples were collected from participants and analyzed for a range of phthalate metabolites, including high molecular weight (HMW) phthalates such as mono(carboxynonyl) phthalate (MCNP), mono(carboxyoctyl) phthalate (MCOP), mono-2-ethyl-5-carboxypentyl phthalate (MECP), mono-(3-carboxypropyl) phthalate (MCPP), and mono-benzyl phthalate (MBzP). Low molecular weight (LMW) phthalates such as mono-ethyl phthalate (MEP), mono-n-butyl phthalate (MBP), and mono-isobutyl phthalate (MiBP) were also included. Phthalate metabolite concentrations were adjusted for urinary creatinine to account for variations in urine dilution.

### 2.4. Covariates

Several covariates were included in our study to control for potential confounding factors. These variables included age, sex, race, educational level, economic status, body mass index (BMI), smoking, and alcohol consumption. Demographic information was obtained through structured interviews. BMI was calculated based on participants’ physical examination data, and participants were categorized into nonsmokers, former smokers, and current smokers based on survey responses. Alcohol consumption was categorized into light, moderate, and heavy intake.

### 2.5. Statistical analysis

All statistical analyses were performed using Stata 17.0 (StataCorp LLC, College Station) and R software, version 4.3.1 (R Foundation for Statistical Computing, Vienna, Austria). Survey design variables and sample weights were incorporated into all analyses to account for the complex sampling design of NHANES, with MEC weights × 0.25 used as the final weights for the 4 survey cycles from 2011 to 2018. Descriptive statistics were used to summarize the demographic characteristics of the study population. Categorical variables were presented as weighted proportions with 95% confidence intervals (95% CI), while continuous variables were presented as weighted means with standard errors (SE). Multivariable linear regression models were employed to examine the association between CDAI and urinary phthalate concentrations, with separate models constructed for each phthalate metabolite using CDAI as the primary independent variable. Model 1 adjusted only for age and sex, while model 2 further adjusted for all aforementioned covariates.

To explore the differences in the association between CDAI and urinary phthalate concentrations across different age groups and sexes, we conducted subgroup analyses. Building on model 2, participants were stratified into different age groups (<60 years, ≥60 years) and sexes (male and female), followed by separate multivariable linear regression analyses.

To examine potential nonlinear relationships between CDAI and urinary phthalate concentrations, restricted cubic spline (RCS) analyses were performed on statistically significant associations from the primary analysis, based on model 1 and model 2. RCS allows for flexible modeling of nonlinear relationships without imposing a specific functional form. Knots were placed at predefined percentiles of the CDAI distribution, and the results were visualized to provide a comprehensive understanding of the dose–response relationship.

## 3. Results

### 3.1. Characteristics of study participants

The baseline characteristics of the study participants are shown in Table 1. Model 1 included 5819 participants adjusted for age and sex, with a mean age of 47.75 years (SE = 0.29). The mean concentrations of HMW phthalates such as MCNP, MCOP, MECP, MCPP, and MBzP were 4.28 ng/mL, 34.21 ng/mL, 15.51 ng/mL, 4.98 ng/mL, and 8.25 ng/mL, respectively. The mean concentrations of LMW phthalates such as MEP, MBP, and MiBP were 149.62 ng/mL, 16.61 ng/mL, and 12.09 ng/mL, respectively. In model 2, which included 4308 participants adjusted for additional covariates, the mean age was 46.55 years (SE = 0.33). The distributions of sex, race, educational level, socioeconomic status, smoking status, and alcohol intake were similar to those in model 1. The mean concentrations of high molecular weight phthalates such as MCNP, MCOP, MECP, MCPP, and MBzP were 4.33 ng/mL, 35.47 ng/mL, 15.07 ng/mL, 4.99 ng/mL, and 8.2 ng/mL, respectively. The mean concentrations of low molecular weight phthalates such as MEP, MBP, and MiBP were 137.73 ng/mL, 15.86 ng/mL, and 12.27 ng/mL, respectively.

**Table 1:** Weighted baseline characteristics of the participants.

Characteristic	Model 1* (N = 5819)	Model 2† (N = 4308)
Age (yr), mean (SE)	47.75 ± 0.29	46.55 ± 0.33
Sex (%)		
Male	49.59 (47.8, 51.38)	50.45 (48.39, 52.51)
Female	50.41 (48.62, 52.2)	49.55 (47.49, 51.61)
Race (%)		
Non-Hispanic White	66.29 (64.85, 67.7)	68.82 (67.23, 70.36)
Non-Hispanic Black	11.45 (10.76, 12.18)	10.69 (9.93, 11.49)
Mexican American	7.97 (7.34, 8.65)	7.52 (6.82, 8.28)
Non-Hispanic Asian	4.78 (4.39, 5.2)	4.31 (3.9, 4.77)
Hispanic	6.05 (5.5, 6.66)	5.36 (4.76, 6.02)
Other race	3.46 (2.9, 4.12)	3.31 (2.69, 4.07)
Educational level (%)		
Below high school	13.3 (12.37, 14.28)	11.15 (10.19, 12.19)
High school	22.4 (20.92, 23.95)	22.26 (20.56, 24.06)
Some college or AA degree	31.84 (30.22, 33.49)	32.1 (30.25, 34)
College graduate or above	32.41 (30.66, 34.22)	34.49 (32.47, 36.57)
Socioeconomic status (%)		
Low	12.9 (11.98, 13.88)	13.5 (12.41, 14.66)
Moderate	44.09 (42.34, 45.86)	46.28 (44.25, 48.32)
High	35.51 (33.69, 37.38)	40.23 (38.12, 42.36)
BMI (%)		
<25 kg/m^2^	28.89 (27.29, 30.54)	29.25 (27.43, 31.15)
25–30 kg/m^2^	31.81 (30.15, 33.51)	32.21 (30.29, 34.18)
≥30 kg/m^2^	38.75 (37.03, 40.5)	38.54 (36.56, 40.56)
Smoking status (%)		
Never	57.96 (56.18, 59.71)	58.12 (56.08, 60.13)
Former	23.44 (21.93, 25.02)	23.01 (21.29, 24.83)
Current	18.58 (17.28, 19.95)	18.87 (17.39, 20.45)
Alcohol intake (%)		
Low	8.92 (8.05, 9.86)	9.79 (8.77, 10.92)
Moderate	34.49 (32.76, 36.27)	40.56 (38.53, 42.63)
High	42.34 (40.57, 44.12)	49.64 (47.58, 51.71)
HMW phthalates (ng/mL) mean (SE)		
MCNP	4.28 ± 0.36	4.33 ± 0.43
MCOP	34.21 ± 1.54	35.47 ± 1.86
MECP	15.51 ± 0.52	15.07 ± 0.59
MCPP	4.98 ± 0.35	4.99 ± 0.37
MBzP	8.25 ± 0.28	8.2 ± 0.34
LMW phthalates (ng/mL) mean (SE)		
MEP	149.62 ± 10.4	137.73 ± 9.07
MBP	16.61 ± 1.12	15.86 ± 1.08
MiBP	12.09 ± 0.42	12.27 ± 0.51

BMI = body mass index, HMW = high molecular weight, LMW = low molecular weight, MCNP = mono(carboxynonyl)phthalate, MCOP = mono(carboxyoctyl) phthalate, MECP = mono-2-ethyl-5-carboxypentyl phthalate, MCPP = mono-(3-carboxypropyl) phthalate, MBzP = mono-benzyl phthalate, MEP = mono-ethyl phthalate, MBP = mono-n-butyl phthalate, MiBP = mono-isobutyl phthalate, SE = standard error.

* Model 1: adjusted for age, sex.

† Model 2: adjusted for age, sex, race, educational level, socioeconomic status, BMI, smoking status, alcohol intake.

### 3.2. Association between the CDAI and urinary phthalate concentrations

The weighted multivariable linear regression analysis of the relationship between CDAI and urinary phthalate concentrations is presented in Table 2. The results of model 1 were consistent with those of model 2. In model 2, after adjusting for all covariates, CDAI was significantly associated with lower concentrations of HMW phthalates MCOP (β = −0.724, 95% CI: −1.454, −0.006) and MECP (β = −0.297, 95% CI: −0.618, −0.091), and showed a significant negative correlation with MBzP (β = −0.212, 95% CI: −0.353, −0.071). For LMW phthalates, CDAI was significantly associated with lower MiBP concentrations (β = −0.136, 95% CI: −0.318, −0.046).

**Table 2:** The weighted multivariate linear regression analysis of the relationship between the composite dietary antioxidant index and urinary phthalate concentrations.

HMW phthalates	CDAI	Model 1† β (95% CI)	Model 2‡ β (95% CI)
MCNP	Continuous	−0.022 (−0.226, 0.181)	−0.074 (−0.330, 0.182)
	Q1	Reference	Reference
	Q2	−0.346 (−2.294, 1.603)	−1.029 (−3.420, 1.362)
	Q3	−0.125 (−2.019, 1.770)	−0.403 (−2.913, 2.107)
MCOP	Continuous	−**0.657** (−**1.293, −0.020**)*	−**0.724** (−**1.454, −0.006**)*
	Q1	Reference	Reference
	Q2	−1.890 (−9.894, 6.115)	−1.740 (−12.802, 9.323)
	Q3	−5.737 (−12.398, 0.924)	−6.612 (−14.985, 1.761)
MECP	Continuous	−**0.285** (−**0.561, −0.008**)*	−**0.297** (−**0.618, −0.091**)*
	Q1	Reference	Reference
	Q2	−1.711 (−4.323, 0.902)	−0.682 (−3.745, 2.380)
	Q3	−**2.589** (−**5.167, −0.011**)*	−2.306 (−5.250, 0.638)
MCPP	Continuous	−0.133 (−0.362, 0.095)	−0.115 (−0.343, 0.114)
	Q1	Reference	Reference
	Q2	−1.189 (−3.171, 0.792)	−0.624 (−2.471, 1.222)
	Q3	−1.114 (−3.372, 1.143)	−0.834 (−2.847, 1.179)
MBzP	Continuous	−**0.297** (−**0.423, −0.172**)***	−**0.212** (−**0.353, −0.071**)**
	Q1	Reference	Reference
	Q2	−**2.413** (−**3.978, −0.847**)**	−1.499 (−3.402, 0.404)
	Q3	−**3.150** (−**4.556, −1.744**)***	−**2.195** (−**3.843, −0.547**)**
*LMW phthalates*	*CDAI*		
MEP	Continuous	0.955 (−4.369, 6.279)	0.924 (−3.250, 5.098)
	Q1	Reference	Reference
	Q2	−7.164 (−53.516, 39.189)	−8.013 (−54.840, 38.814)
	Q3	−8.845 (−66.678, 48.987)	−13.343 (−57.644, 30.959)
MBP	Continuous	−0.195 (−0.802, 0.411)	0.052 (−0.468, 0.572)
	Q1	Reference	Reference
	Q2	−1.655 (−8.293, 4.984)	1.396 (−3.089, 5.882)
	Q3	−4.506 (−10.612, 1.600)	−1.376 (−4.629, 1.877)
MiBP	Continuous	−**0.176** (−**0.308, −0.050**)*	−**0.136** (−**0.318, −0.046**)*
	Q1	Reference	Reference
	Q2	−**1.665** (−**3.125, −0.206**)*	−0.870 (−2.653, 0.912)
	Q3	−1.366 (−3.354, 0.623)	−0.406 (−2.640, 1.829)

BMI = body mass index, CI = confidence interval, HMW = high molecular weight, LMW = low molecular weight, MCNP = mono(carboxynonyl)phthalate, MCOP = mono(carboxyoctyl) phthalate, MECP = mono-2-ethyl-5-carboxypentyl phthalate, MCPP = mono-(3-carboxypropyl) phthalate, MBzP = mono-benzyl phthalate, MEP = mono-ethyl phthalate, MBP = mono-n-butyl phthalate, MiBP = mono-isobutyl phthalate.

Bold values indicate statistical significance.

† Model 1: adjusted for age, sex.

‡ Model 2: adjusted for age, sex, race, educational level, socioeconomic status, BMI, smoking status, alcohol intake.

* *P* < .05.

** *P* < .01.

*** *P* < .001.

### 3.3. Subgroup analysis

Subsequently, we conducted subgroup analyses on the significant results from the multivariable linear regression of model 2, stratified by age and gender, as shown in Tables 3 and 4. Age-stratified analyses revealed significant associations between CDAI and lower concentrations of MCOP (β = −1.116, *P* = .022), MECP (β = −0.486, *P* = .032), and MBzP (β = −0.268, *P* = .002) among participants aged 60 years and above; no significant associations were found between CDAI and MiBP. In contrast, no significant associations were observed among participants under 60 years of age. Gender-stratified subgroup analyses indicated significant associations between CDAI and concentrations of MCOP (β = −0.887, *P* = .045), MECP (β = −0.558, *P* = .039), MBzP (β = −0.255, *P* = .003), and MiBP (β = −0.323, *P* = .010) among male participants. In contrast, no significant associations were found between CDAI and these phthalates among female participants.

**Table 3:** Association between the composite dietary antioxidant index and urinary phthalate concentrations after subgroup analysis by age.*

	<60 yr		≥60 yr	
Outcomes	β (95% CI)	*P* value	β (95% CI)	*P* value
MCOP	−0.585 (−1.471, 0.302)	.196	−**1.116** (−**2.069, −0.163**)	.022
MECP	−0.226 (−0.618, 0.167)	.259	−**0.486** (−**0.930, −0.041**)	.032
MBzP	−0.145 (−0.379, 0.089)	.225	−**0.268** (−**0.440, −0.097**)	.002
MiBP	−0.128 (−0.342, 0.087)	.243	−0.177 (−0.501, 0.148)	.285

BMI = body mass index, CI = confidence interval, MCOP, mono(carboxyoctyl) phthalate; MECP, Mono-2-ethyl-5-carboxypentyl phthalate; MBzP, mono-benzyl phthalate; MiBP, mono-isobutyl phthalate.

* Adjusted for sex, race, educational level, socioeconomic status, BMI, smoking status, alcohol intake. Bold values indicate statistical significance.

**Table 4:** Association between the composite dietary antioxidant index and urinary phthalate concentrations after subgroup analysis by sex.*

	Male		Female	
Outcomes	β (95% CI)	*P* value	β (95% CI)	*P* value
MCOP	−**0.887** (−**1.964, −0.034**)	.045	−0.643 (−1.564, 0.278)	.171
MECP	−**0.558** (−**1.063, −0.053**)	.039	0.112 (−0.228, 0.452)	.519
MBzP	−**0.255** (−**0.422, −0.089**)	.003	−0.146 (−0.397, 0.104)	.253
MiBP	−**0.323** (−**0.568, −0.078**)	.010	0.124 (−0.141, 0.388)	.360

BMI = body mass inde, CI = confidence interval, MCOP = mono(carboxyoctyl) phthalate, MECP = mono-2-ethyl-5-carboxypentyl phthalate, MBzP = mono-benzyl phthalate, MiBP = mono-isobutyl phthalate.

* Adjusted for age, race, educational level, socioeconomic status, BMI, smoking status, alcohol intake. Bold values indicate statistical significance.

### 3.4. RCS analysis

**Figure 2.**
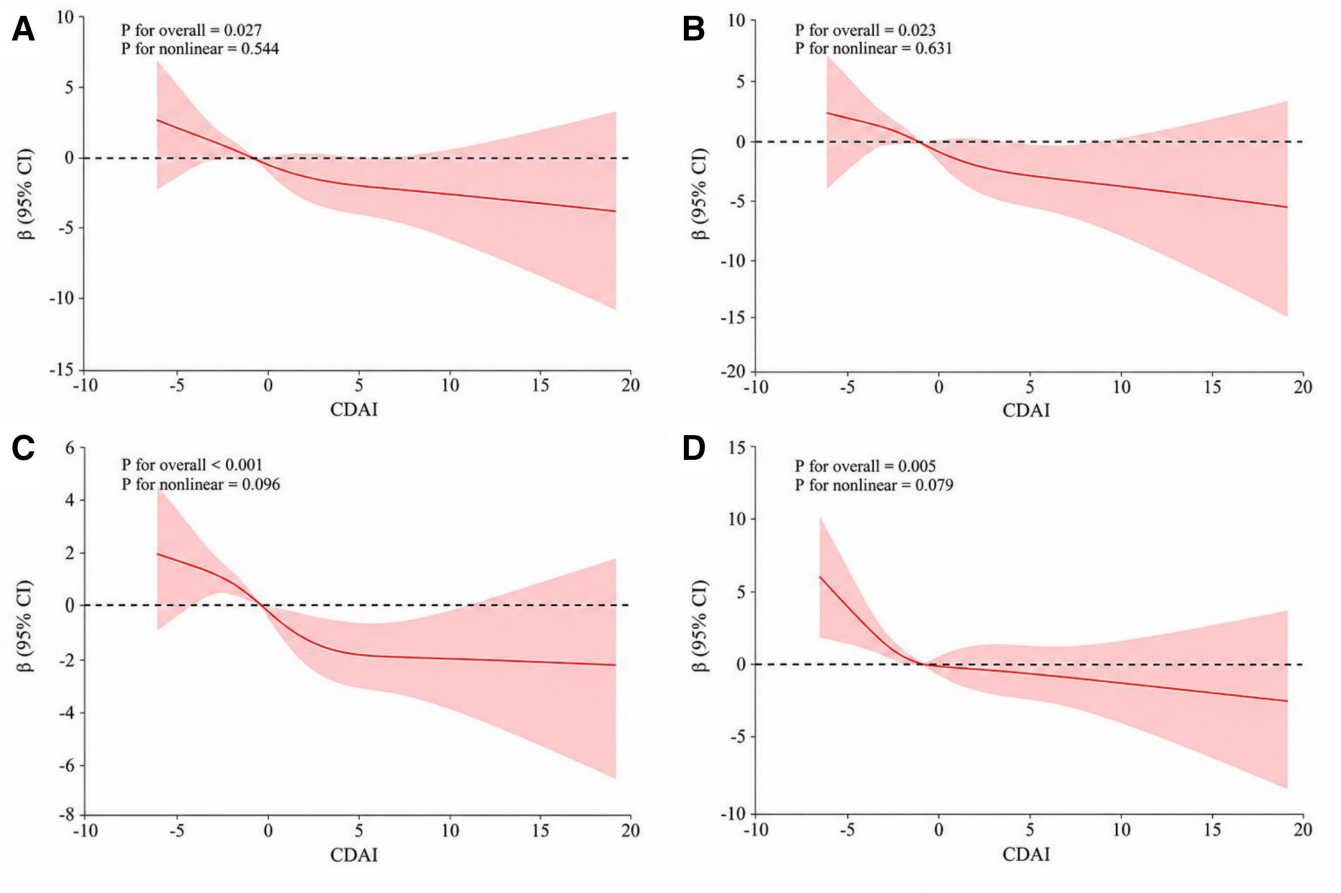
Weighted RCS analysis of the association between the composite dietary antioxidant index and urinary phthalate concentrations. (A) RCS curve of the association between CDAI and MCOP; (B) RCS curve of the association between CDAI and MECP; (C) RCS curve of the association between CDAI and MBzP; (D) RCS curve of the association between CDAI and MiBP. The model was adjusted for age, sex. CDAI = Composite Dietary Antioxidant Index, CI = confidence interval, MCOP = mono(carboxyoctyl) phthalate, MECP = mono-2-ethyl-5-carboxypentyl phthalate, MiBP = mono-isobutyl phthalate, RCS = restricted cubic spline.

**Figure 3.**
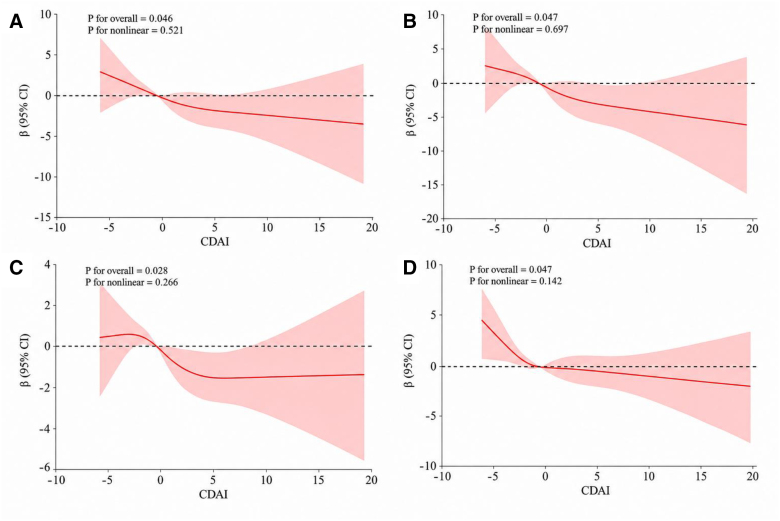
Weighted RCS analysis of the association between the composite dietary antioxidant index and urinary phthalate concentrations. (A) RCS curve of the association between CDAI and MCOP; (B) RCS curve of the association between CDAI and MECP; (C) RCS curve of the association between CDAI and MBzP; (D) RCS curve of the association between CDAI and MiBP. The model was adjusted for age, sex, race, educational level, socioeconomic status, BMI, smoking status, alcohol intake. CDAI = Composite Dietary Antioxidant Index, CI = confidence interval, MBzP = mono-benzyl phthalate, MCOP = mono(carboxyoctyl) phthalate, MECP = mono-2-ethyl-5-carboxypentyl phthalate, MiBP = mono-isobutyl phthalate, RCS = restricted cubic spline.

The purpose of conducting RCS analysis is to explore the potential nonlinear relationship between CDAI and urinary phthalate concentrations. We performed weighted RCS analysis based on model 1 (Fig. 2). The results showed that CDAI was negatively correlated with MCOP, MECP, MBzP, and MiBP (*P* for overall < .05), and the relationship was linear (*P* for nonlinear > .05). Subsequently, we conducted weighted RCS analysis on model 2 after adjusting for all covariates (Fig. 3). The results indicated that CDAI remained negatively correlated with MCOP, MECP, MBzP, and MiBP (*P* for overall < .05), and the relationship remained linear (*P* for nonlinear > .05).

## 4. Discussion

This study utilized NHANES 2011–2018 data to analyze the association between CDAI and urinary phthalate concentrations using weighted multivariable linear regression. Our results indicate a significant negative correlation between CDAI and several HMW phthalates, including MCOP, MECP, and MBzP, as well as 1 LMW phthalate, MiBP. Notably, this association was more pronounced for HMW phthalates. Subgroup analysis revealed that these associations were significant among participants aged 60 years and older, and among male participants, but not significant among younger or female participants. Weighted RCS analysis of both models demonstrated a linear negative relationship between CDAI and MCOP, MECP, MBzP, and MiBP.

Phthalates, as endocrine-disrupting chemicals (EDCs), have the potential to mimic or interfere with normal hormonal functions. Studies have demonstrated that phthalates reduce the expression of certain downstream genes of the peroxisome proliferator-activated receptor (PPAR), a function that may play a critical role in adult aldosterone biosynthesis.^[21]^ Additionally, phthalates can act as receptor antagonists to directly inhibit 11β-hydroxylase, thereby reducing cortisol synthesis, or act as agonists to modulate AT1 receptors or 11β-hydroxylase type 2 receptors, stimulating aldosterone biosynthesis.^[22]^ Exposure to phthalates may result in alterations in sex hormone levels,^[23]^ thereby impacting the development of the reproductive system. Phthalates can cause reproductive toxicity by disrupting the hypothalamic-pituitary-gonadal (HPG) axis.^[24]^ Studies indicate that phthalates may increase the risk of ovarian aging, leading to diminished ovarian reserve and consequently affecting fertility.^[25]^ A prospective cohort study involving 139 pregnant women found that phthalates may influence hormonal changes during pregnancy and contribute to postpartum depression (PPD).^[26]^ The research conducted by Matilde Lærkeholm Müller et al revealed that exposure to phthalates could alter the concentrations of male reproductive hormones in male infants during minipuberty.^[27]^ Our findings indicate that dietary antioxidants are associated with lower urinary phthalate concentrations, suggesting that these antioxidants may mitigate health damages caused by phthalate exposure. Studies show that healthier dietary patterns, such as the consumption of organic foods, are linked to reduced urinary phthalate metabolites.^[28-31]^

Notably, among the 8 phthalate metabolites included in the study, CDAI exhibited a more pronounced negative correlation with HMW phthalates. The significant negative correlation between CDAI and HMW phthalates, such as MCOP and MECP, is likely attributable to the distinct toxicokinetic properties of HMW and LMW phthalates. Compared to LMW phthalates, HMW phthalates generally have longer half-lives and a greater tendency for bioaccumulation in the body.^[32]^ In the subgroup analysis, we observed a significant association between CDAI and urinary phthalate concentrations among participants aged 60 and above. This may be attributed to age-related differences in metabolism and antioxidant capacity.^[33]^ Elderly individuals typically exhibit reduced metabolic efficiency and lower levels of endogenous antioxidants, rendering them more susceptible to oxidative stress and toxicants such as phthalates.^[34]^ Consequently, diets rich in antioxidants may offer a more pronounced association for this demographic. The inverse correlation between CDAI and urinary phthalate concentrations is more pronounced in men than in women, potentially due to differences in dietary patterns, hormonal regulation, and body composition. Studies have indicated that the male reproductive system exhibits heightened sensitivity to EDCs such as bisphenols.^[35,36]^ Moreover, men and women may exhibit distinct dietary and behavioral patterns that influence their antioxidant intake. Additionally, the differential hormonal effects of phthalates could result in varying impacts between genders.^[37]^ Furthermore, the gender-specific distribution of body fat,^[38]^ which varies between men and women, may influence the storage and mobilization of lipophilic substances such as phthalates.

The NHANES employed a sophisticated sampling design, with a large sample size, diverse data, and strong representativeness, thereby enhancing the reliability of the conclusions. Utilizing weighted multivariate linear regression and RCS analysis robustly explores both linear and potential nonlinear relationships between CDAI and urinary phthalate concentrations. The inverse associations observed in this study may reflect differences in dietary patterns rather than a direct biological effect of antioxidants on phthalate metabolism. Higher CDAI may indicate greater intake of fresh fruits, vegetables, and minimally processed foods, which may involve less contact with plastic packaging and food-processing materials. Therefore, CDAI may serve not only as an indicator of antioxidant intake but also as a proxy for healthier dietary behaviors associated with lower phthalate exposure.

However, several limitations should be noted. Because this study used a cross-sectional design, the observed inverse associations between CDAI and urinary phthalate metabolites should not be interpreted as causal. Individuals with higher CDAI scores may also have healthier lifestyles, consume less processed or packaged food, or have higher socioeconomic status, all of which may be related to lower phthalate exposure. Therefore, reverse causation and residual confounding cannot be excluded. Residual confounding remains possible because several important determinants of phthalate exposure were not fully captured, including consumption of processed foods, food packaging materials, takeout or delivery food use, and contact with consumer products. Physical activity and broader dietary quality may also confound the association between CDAI and urinary phthalate concentrations. Although urinary creatinine was used to correct for urine dilution, alternative approaches such as specific gravity correction were not available in the present analysis and should be considered in future studies.

## 5. Conclusions

In conclusion, higher CDAI was associated with lower urinary concentrations of several phthalate metabolites, particularly HMW phthalates, in U.S. adults from NHANES 2011–2018. These findings should be interpreted as associations rather than causal effects because of the cross-sectional study design and potential residual confounding. Future longitudinal studies incorporating detailed dietary sources, food packaging exposure, physical activity, supplement use, and alternative urine dilution correction methods are needed to confirm these findings.

## Acknowledgments

We appreciate the data provided by the NHANES database.

## Author contributions

**Conceptualization:** Yongjiao Yang, Pan Ding, Jiancheng Pan, Yuhong Feng, Zhongcheng Xin, Changli Wu.

**Data curation:** Yongjiao Yang, Pan Ding, Jiancheng Pan, Yuhong Feng, Zhongcheng Xin, Changli Wu.

**Formal analysis:** Yongjiao Yang, Pan Ding, Jiancheng Pan, Yuhong Feng, Zhongcheng Xin, Changli Wu.

**Funding acquisition:** Yongjiao Yang, Pan Ding, Jiancheng Pan, Yuhong Feng, Zhongcheng Xin, Changli Wu.

**Investigation:** Yongjiao Yang, Jiancheng Pan, Yuhong Feng, Zhongcheng Xin, Changli Wu.

**Writing – original draft:** Yongjiao Yang, Yuhong Feng, Zhongcheng Xin, Changli Wu.

**Writing – review & editing:** Yongjiao Yang, Yuhong Feng, Zhongcheng Xin, Changli Wu.
